# Evaluation of 6-substituted coumarins as SARS-CoV-2 main protease inhibitors: enzymatic, cellular, and computational insights

**DOI:** 10.1039/d6ra07298d

**Published:** 2026-10-05

**Authors:** Ashraf K. El-Damasy, Md Sofequl Islam Mukim, Sangeun Jeon, Mahmoud A. El Hassab, Suliman A. Almahmoud, Eun-Kyoung Bang, Wagdy M. Eldehna, Dae-Geun Song, Seungtaek Kim, Gyochang Keum

**Affiliations:** a Biomedical Research Division, Korea Institute of Science and Technology (KIST) Seoul 02792 Republic of Korea ph_karem2000@mans.edu.eg gkeum@kist.re.kr; b Department of Medicinal Chemistry, Faculty of Pharmacy, Mansoura University Mansoura 35516 Egypt; c Center for Natural Product Systems Biology, KIST Gangneung Institute of Natural Products Gangneung 25451 Republic of Korea dsong82@kist.re.kr; d Natural Product Applied Science, KIST School, University of Science and Technology Gangneung 25451 Republic of Korea; e Zoonotic Virus Laboratory, Institut Pasteur Korea Seongnam Republic of Korea seungtaek.kim@ip-korea.org; f Department of Medicinal Chemistry, Faculty of Pharmacy, King Salman International University (KSIU) South Sinai 46612 Egypt; g Department of Pharmaceutical Chemistry and Pharmacognosy, College of Pharmacy, Qassim University Buraydah 51452 Saudi Arabia; h Department of Pharmaceutical Chemistry, Faculty of Pharmacy, Kafrelsheikh University Kafrelsheikh P.O. Box 33516 Egypt wagdy2000@gmail.com; i Division of Bio-Medical Science and Technology, KIST School, University of Science and Technology Seoul 02792 Republic of Korea

## Abstract

The SARS-CoV-2 main protease (M^pro^) plays a crucial role in viral replication, making it an attractive target for antiviral drug development. This study evaluates the inhibitory potential of nineteen 6-substituted coumarin derivatives (1a–p and 2a–c) against M^pro^ using a FRET-based enzymatic assay. Among them, the chlorinated derivative 1f (AKE-9306) exhibited the highest potency with an IC_50_ value of 61.77 µM. Enzyme kinetics analysis through Michaelis–Menten and Lineweaver–Burk plots revealed that compound 1f may act through a mixed-type inhibition mechanism with a preference for the free enzyme. In cellular assays, 1f showed significant antiviral efficacy (IC_50_ = 16.02 µM, CC_50_ >50 µM, selectivity index >3.12). Molecular docking and dynamics simulations confirmed its stable interaction within the M^pro^ active site, supported by MM-PBSA calculations. These findings identify compound 1f as a hit coumarin-based scaffold for further optimization toward improved SARS-CoV-2 M^pro^ inhibition and antiviral activity.

## Abstract 2

The SARS-CoV-2 main protease (M^pro^) plays a crucial role in viral replication, making it an attractive target for antiviral drug development.

## Graphical abstract

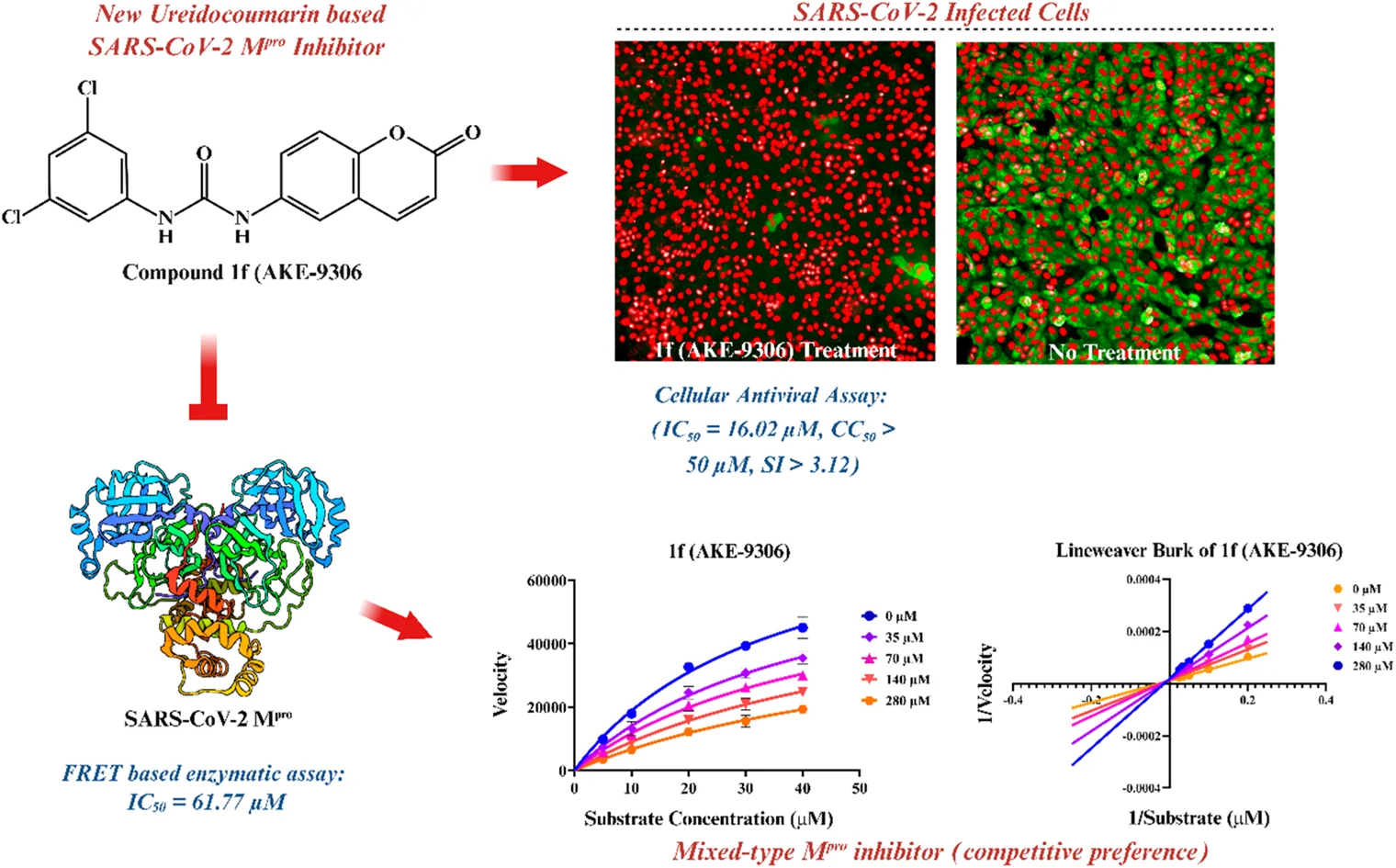

## 1. Introduction

The global outbreak of COVID-19, caused by the new coronavirus SARS-CoV-2, started in late 2019 and rapidly became a pandemic, posing major challenges to public health systems.^1–3^ This positive-sense single-stranded RNA virus relies on a complex collection of enzymes to copy its RNA genome.^4^ As of September 2026, confirmed deaths worldwide stand at 7 116 408, according to the World Health Organization (WHO) and aggregated public health databases.^5^ The far-reaching impacts of SARS-CoV-2 go well beyond health issues, causing unprecedented economic and societal disruptions.^6^ Despite the tremendous speed of vaccine development, the continuing threat of the virus clearly indicates the urgent need for potent antiviral therapies. Such therapies are required for both the treatment of current infections and, importantly, reducing the possibility of the virus assuming pandemic conditions again 7.

One of the principal focuses of activity in the design of antiviral drugs has been the main protease of SARS-CoV-2, otherwise known as M^pro^, and sometimes referred to as 3-chymotrypsin-like protease (3CL^pro^).^8–11^ The enzyme plays a crucial role in viral survival and propagation by cleaving the polyproteins pp1a and pp1ab. This proteolytic processing by M^pro^ liberates 13 of the 16 non-structural proteins that are required for viral replication and transcription.^12,13^ Inhibition of this protease could severely impair the virus's ability to replicate, offering a promising avenue for treating COVID-19 and mitigating its long-term impacts.^14,15^

Carbonic anhydrase (CA) enzymes have been suggested as key players in the pathophysiology underlying COVID-19, mainly because of their role in acid–base equilibrium and hypoxia, which offers potential therapeutic targets.^16,17^ Dysregulation of CA, along with other biomolecules, has been implicated in the pathogenesis of disease complications, such as respiratory acidosis and pulmonary edema.^18^ Upregulation of CA activity in patients with COVID-19 is significantly higher in the post-COVID setting than in the acute setting.^16,19^ Clinical manifestations of COVID-19, such as decreased arterial oxygen, reduced CO_2_ levels, hypoxia, and lung issues, resemble high-altitude pulmonary edema (HAPE), which is characterized by hypoxia, tachypnea, lowered arterial oxygen levels, and affected lungs.^20,21^ Notably, acetazolamide, a CA inhibitor used in HAPE treatment, has been proposed as a therapeutic candidate for COVID-19 due to its ability to alleviate hypoxic pulmonary vasoconstriction and improve ventilation in HAPE patients.^19,20,22,23^ Furthermore, CA IX expression is upregulated by HIF-1 under conditions of low oxygen availability, which can lead to extracellular acidification, creating a pro-inflammatory milieu and thus exacerbating disease severity.^24–26^ Celecoxib displayed potent inhibitory activity in the nanomolar range towards CA IX (*K*_i_ = 16 nM).^27^ In addition, it is believed to improve disease-free survival in COVID-19 patients.^28^ Dexamethasone, a cornerstone in the treatment of COVID-19, also downregulates CA IX expression through HIF-1α-dependent pathways, further underlining the interplay between CA and COVID-19 therapeutics.^29^

The 1,3-biarylurea-based compounds have been a fundamental part of the chemical space of compounds exhibiting excellent activity against CA IX,^30^ such as SLC-0111,^31^ as well as benzofuran and 2,3-dihydro-1,4-dioxin-based SLC-0111 analogs I and II, respectively.^32,33^ Recently, compounds bearing the 1,3-biarylurea scaffold have emerged, exhibiting notable activity against SARS-CoV-2, represented by compound III with an IC_50_ value of 3.97 µM.^34^ Of interest, the Kumar N. *et al.* study disclosed compound IV exhibiting sub-micromolar cellular activity against SARS-CoV-2,^35^Fig. 1.

**Fig. 1:**
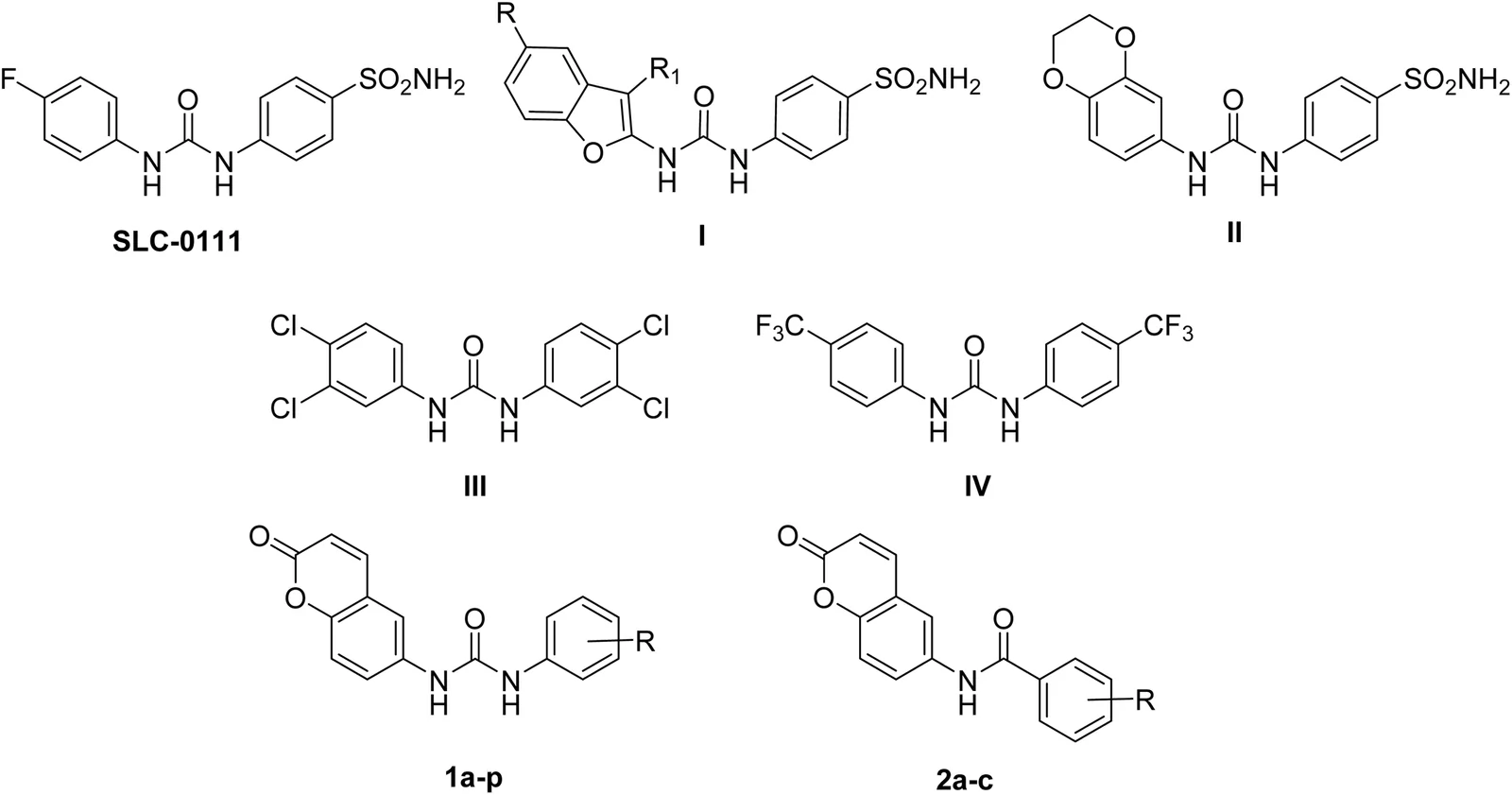
Chemical structures of compounds SLC-0111 and I–IV (with effective inhibitory activity towards CA IX or SARS-CoV-2) along with the currently investigated coumarin derivatives 1a–p, and 2a–c.

In light of the continuous emergence of SARS-CoV-2 variants, drug repurposing and scaffold-hopping strategies applied to privileged scaffolds represent highly efficient approaches for rapidly identifying novel antiviral candidates.^36,37^ Coumarin and arylurea architectures are widely recognized as substantial scaffolds with considerable anticancer and antiviral activities. Our previous work established that these coumarin-incorporated arylurea derivatives exhibit potent inhibitory properties against human CAs and DDR1 kinases.^38,39^ Because multi-targeting drug discovery often leverages privileged frameworks that can adapt to different enzymatic pockets through flexible binding modes, we hypothesized that combining a coumarin core with arylurea could successfully occupy the sub-pockets of the SARS-CoV-2 main protease (M^pro^). Specifically, the spacious and flexible nature of the arylurea-coumarin derivatives allows them to project into the catalytic cleft of M^pro^, engaging key catalytic and structural residues such as the oxyanion loop and catalytic dyad components (*e.g.*, Cys145, His41, and Glu166) similar to how other privileged heterocyclic scaffolds, including coumarins and biaryl cores, have been successfully cross-screened and optimized as non-covalent M^pro^ inhibitors.^40–43^

Driven by this hypothesis and building upon our previous work where these coumarin-incorporated arylurea (1a–p) and benzamide (2a–c) derivatives were characterized as selective carbonic anhydrase and DDR1 inhibitors,^38,39^ we sought to explore their potential *via* drug repurposing against the SARS-CoV-2 main protease M^pro^. In this study, the focused in-house library was subjected to comprehensive enzymatic screening, multi-dose kinetics, live cellular antiviral evaluation, and molecular modeling to validate compound 1f as a promising hit scaffold for further structural optimization.

## 2. Results and discussion

### 2.1. Biological evaluations

#### 2.1.1. Primary screening against SARS-CoV-2 M^pro^

The catalytic competence of the purified SARS-CoV-2 M^pro^ enzyme was confirmed by FRET-based kinetic analysis, which yielded an apparent *K*_m_ of 29.05 µM and a *K*_cat_ of 1.68 s^−1^ (SI Fig. S1A), values comparable to those reported for the same substrate (F. Li *et al.*, 2023). The biological evaluation was then initiated by conducting a primary screening of the 19 coumarin-based derivatives (1a–p and 2a–c) against the SARS-CoV-2 M^pro^ protein. The primary screening was performed using a FRET assay at a uniform concentration of 100 µM, with GC376, Boceprevir, and Baicalein as positive controls. Interestingly, the inhibitory activities of the tested molecules against M^pro^ revealed a wide range between 0.1% and 65.9% (Table 1).

**Table 1:** Mean inhibition percentages of the 19 coumarin-based derivatives (1a–p and 2a–c) against SARS-CoV-2 M^pro^ at 100 µM using the FRET assay

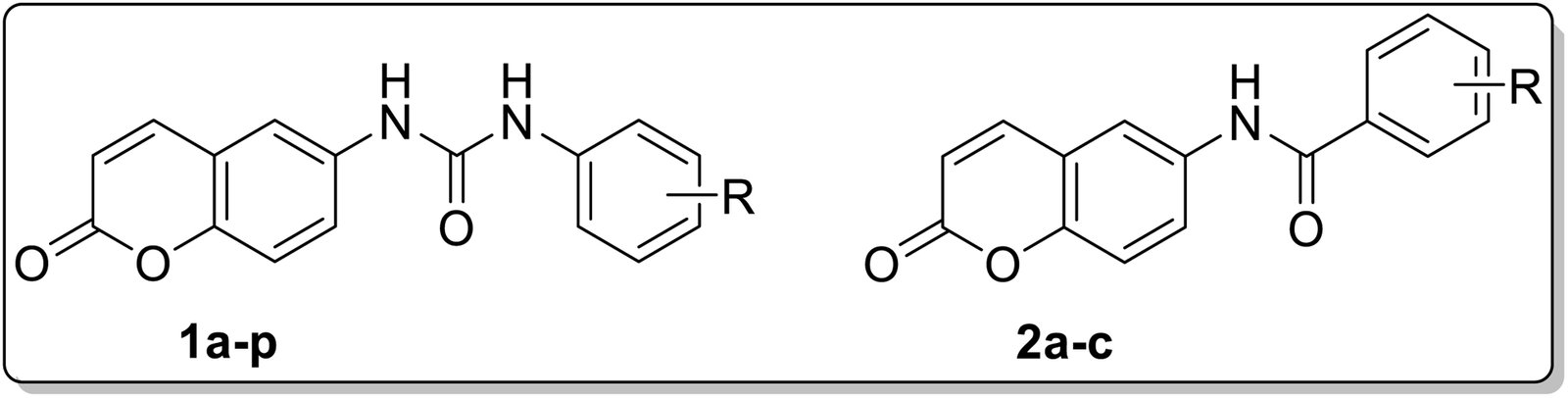		
Compound	R	Mean inhibition%
1a	4-Cl-3-CF_3_	3.0
1b	3,5-(CF_3_)_2_	7.4
1c	3-Cl-4-F	36.7
1d	3-CF_3_	31.9
1e	4-CF_3_	65.9
1f	3,5-Di-Cl	56.7
1g	2,4-Di-Cl	12.0
1h	4-F	32.8
1i	4-Br	7.7
1j	4-*n*-Butyl	15.6
1k	3,5-Di-CH_3_	33.5
1l	4-Butoxy	31.6
1m	4-Cl-2-CH_3_	0.1
1n	2-Cl-6-CH_3_	4.8
1o	2,4-Di-F	14.5
1p	2,6-Di-Br-4-F	18.2
2a	4-Cl-3-CF_3_	22.2
2b	3,5-(CF_3_)_2_	0.9
2c	2,4-Di-F	0.7
GC376	—	100.0
Boceprevir	—	88.0
Baicalein	—	100.1

Among the various derivatives, the trifluoromethylated compound 1e was the most potent inhibitor, with an inhibition rate of 65.9%. The chlorinated derivative 1f (3,5-dichloro) was also shown to be considerably effective, with an inhibition percentage of 56.7%. Meanwhile, compounds 1c–d, 1h, and 1k–l exhibited moderate inhibition, with percentages of inhibition ranging between 31.6% and 36.7%. The remaining compounds (1a–b, 1g, 1i–j, 1m–p, and 2a–c) did not demonstrate any significant activity, as their mean inhibition percentages ranged from 0.1% to 22.2%.

#### 2.1.2. Multiple-dose screening against SARS-CoV-2 M^pro^

Derivatives exhibiting mean inhibition percentages greater than 50% (compounds 1e and 1f) in the primary screening assay were selected for further evaluation to determine their IC_50_ values against the M^pro^. Enzyme activity was assessed at dose-dependent concentrations of each compound to characterize their inhibitory potency. The assay was validated using baicalein as a positive control (IC_50_ = 2.13 µM), which aligns with previously reported values (SI Fig. S1B).

Compound 1e exhibited significant inhibition only at the highest concentration tested, with minimal activity observed at lower concentrations; consequently, its IC_50_ could not be reliably determined. In contrast, its chlorinated analog, 1f, moderately suppressed M^pro^ activity, yielding an IC_50_ of 61.77 µM (SI Fig. S1C). These findings suggest that chlorination at the corresponding position may enhance inhibitory efficacy against M^pro^. It is worth noting that although this activity level is modest compared to the most advanced M^pro^ inhibitors, it falls within the range commonly considered acceptable for hit-stage compounds and provide a basis for future structure–activity refinement.

#### 2.1.3. M^pro^ kinetics analysis

To elucidate the inhibition mechanism of compound 1f, initial-velocity data were fitted by global nonlinear regression to competitive, noncompetitive, uncompetitive, and mixed-inhibition models. The mixed-inhibition model best described the data, yielding an inhibition constant (*K*_i_) of 85.9 µM and an *α* value of 3.5 (Table 2 and Fig. 2). An *α* value greater than unity indicates preferential binding to the free enzyme over the enzyme–substrate complex; the finite value obtained here is therefore consistent with mixed-type inhibition showing a competitive preference, rather than with purely competitive inhibition. These results indicate that 1f is a moderate M^pro^ inhibitor that acts principally, though not exclusively, by interfering with substrate binding.

**Table 2:** Summary of Michaelis–Menten and Lineweaver–Burk plot results from enzyme kinetics assays of 1f

Compound	Inhibition constant, *K*_i_ (µM)	Alpha (*α*)	Goodness of fit (*R*^2^)	Mechanism of inhibition
1f	85.9	3.5	0.98	Mixed-type inhibition (competitive preference)

**Fig. 2:**
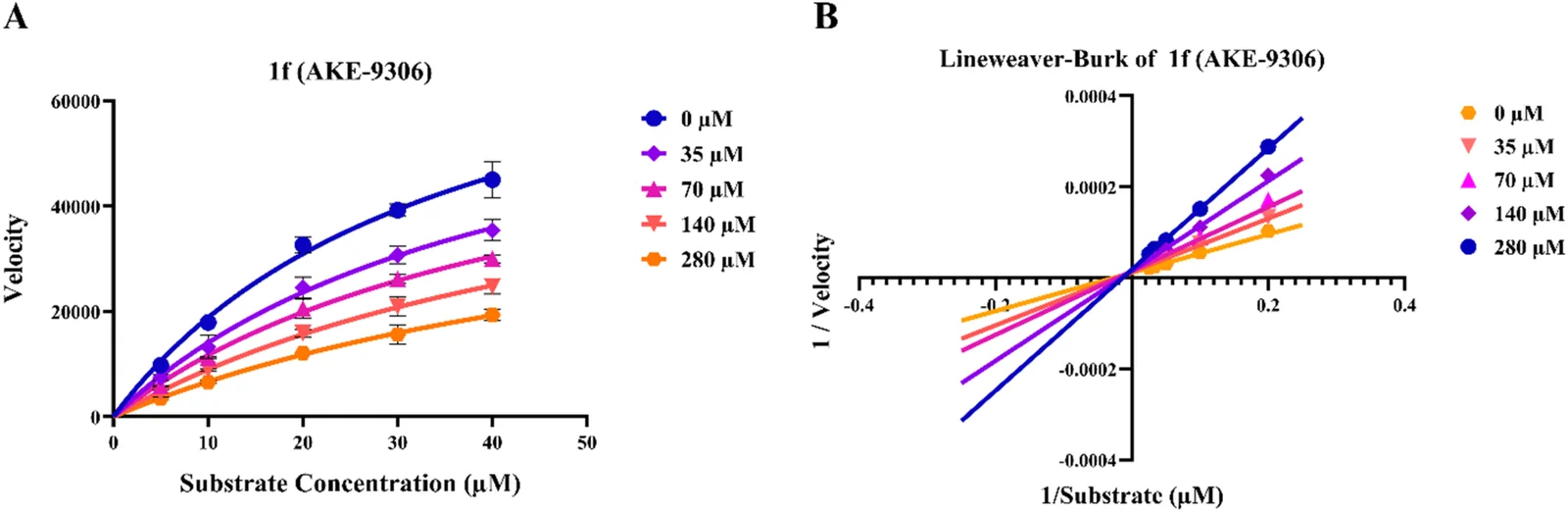
Michaelis–Menten Kinetics and Lineweaver–Burk Plots of SARS-CoV-2 M^pro^ and 1f (AKE-9306). The initial reaction velocity was evaluated as a function of substrate concentration across various inhibitor concentrations. Data were analyzed using nonlinear regression based on the Michaelis–Menten model. Michaelis–Menten plots are shown in (A), and the corresponding Lineweaver–Burk plot is presented in (B).

#### 2.1.4. Cell viability analysis of AKE-9306 (1f) in IMR-90 normal human lung fibroblasts

The cytotoxicity of 1f was evaluated in IMR-90 normal human lung fibroblast cells after 24 h of treatment. As shown in Fig. 3, 1f did not markedly reduce cell viability across the tested concentration range of 3.12–100 µM compared with the DMSO-treated control group. Cell viability remained largely maintained even at the highest tested concentration, indicating that 1f exhibited no apparent cytotoxicity in normal human lung fibroblast cells under these experimental conditions. These findings suggest that the inhibitory activity of AKE-9306 (1f) was not associated with pronounced cytotoxic effects in normal cells at concentrations up to 100 µM, thereby supporting its suitability for further biological evaluation.

**Fig. 3:**
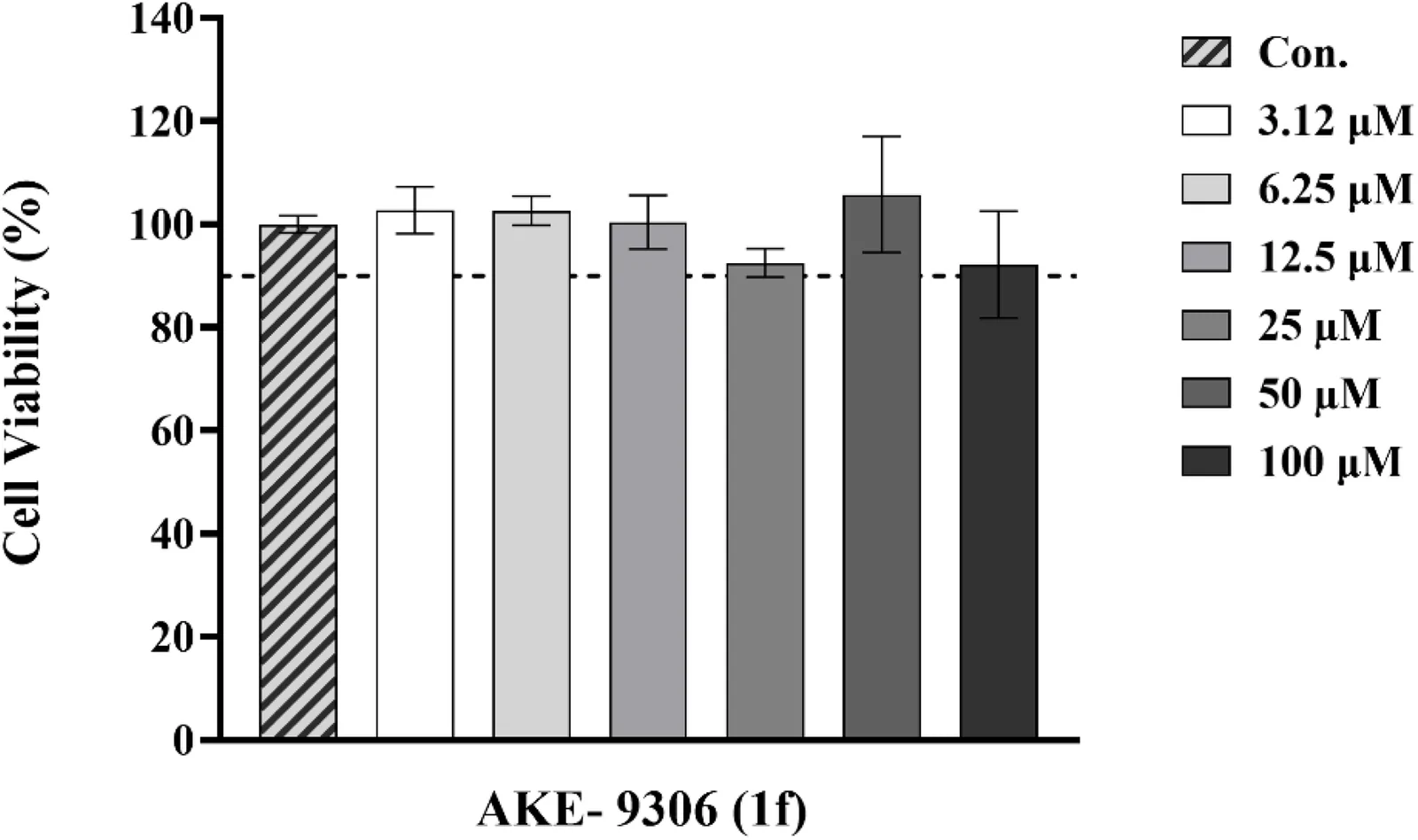
Cytotoxicity of AKE-9306 (1f) in IMR-90 normal human lung fibroblast cells. IMR-90 cells were treated with (1f) at 3.12–100 µM for 24 h, while control cells received an equivalent volume of DMSO. Cell viability was determined using the EZ-Cytox assay and expressed relative to the DMSO-treated control. Data are presented as the mean ± SD.

#### 2.1.5. Live virus assay reveals potent activity of AKE-9306 (1f) against SARS-CoV-2

To assess the antiviral efficacy of 1f, a live SARS-CoV-2 infection assay was conducted in Vero cells. Cells were infected with the ancestral strain of SARS-CoV-2 at a multiplicity of infection (MOI) of 0.1 immediately following treatment with 1f at concentrations of 25 µM and 5 µM. After 24 hours of incubation at 37 °C, viral replication was quantified by immunostaining for the SARS-CoV-2 nucleocapsid (N) protein and nuclear counterstaining, followed by confocal imaging. In the primary screen, 1f showed substantial antiviral activity at 25 µM (68.5% inhibition), while no effect was observed at 5 µM. Importantly, cell viability remained above 80% at both concentrations (SI Table 1).

A subsequent dose–response analysis (Fig. 4A) was performed across a range of concentrations from 50 µM to 0.1 µM. 1f demonstrated concentration-dependent inhibition of viral replication, with an IC_50_ of 16.02 µM, a CC_50_ greater than 50 µM, and a calculated selectivity index (SI) of 3.12. Lopinavir and Remdesivir were included as reference compounds to validate assay performance (Fig. 4B and C). Representative immunofluorescence images (Fig. 4D–K) corroborated these findings, showing a marked reduction in N protein expression at 50 µM, 25 µM, and 12.5 µM relative to mock- and DMSO-treated controls.

**Fig. 4:**
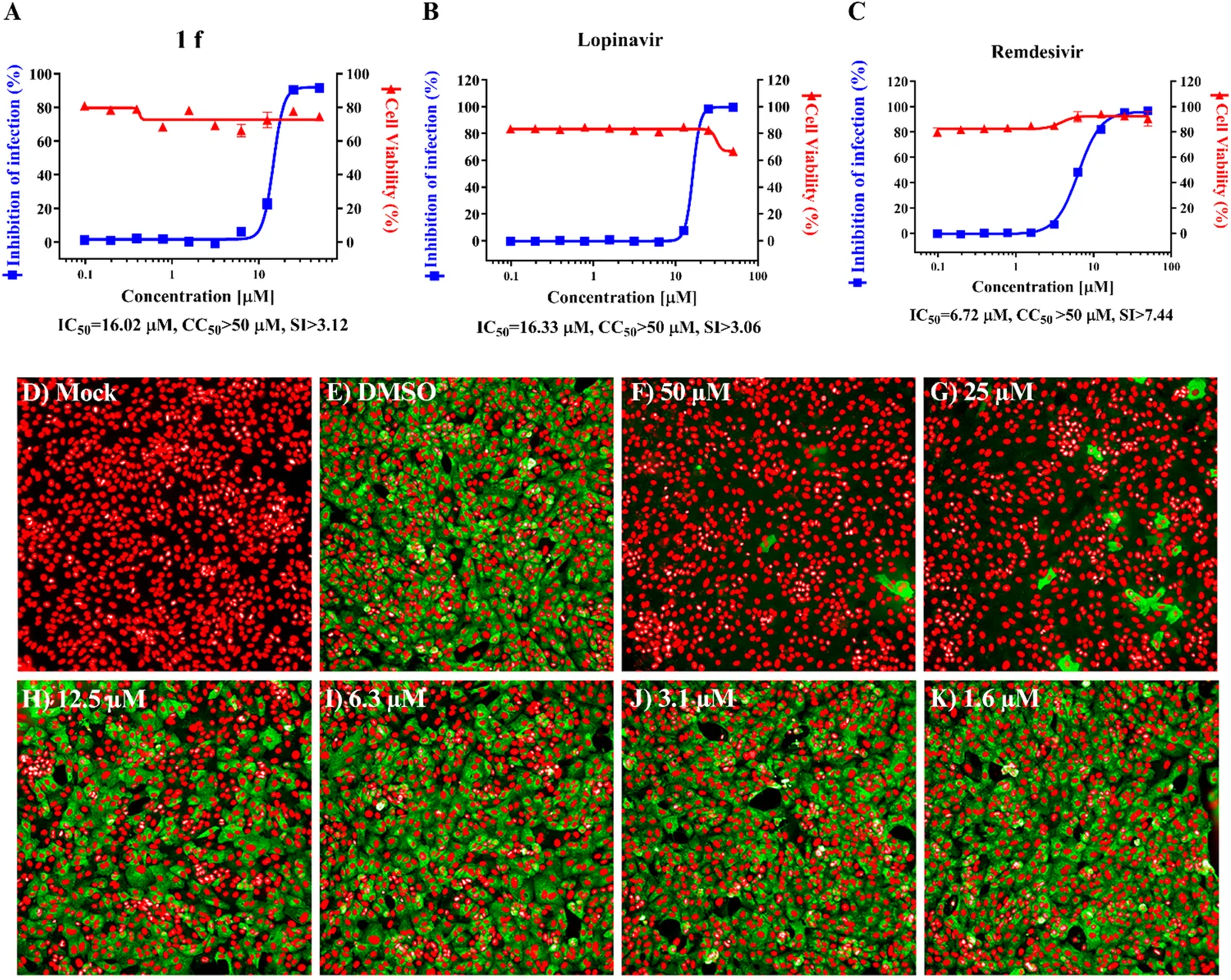
Live virus assay of compound 1f (AKE-9306). Dose–response curves of 1f, Lopinavir, and Remdesivir, respectively (A–C). Representative immunofluorescence images at different concentrations of compound 1f (D–K).

While *in vivo* models remain the gold standard for evaluating antiviral efficacy, *in vitro* testing using human lung cells provides a practical alternative to Vero-based systems when animal models are not readily available. Of interest, the ∼4-fold greater potency of 1f observed in the cellular assay compared to the FRET-based enzymatic assays may reflect enhanced intracellular accumulation, physiologically relevant assay conditions, or potential synergistic modulation of host pathways that facilitate viral replication. While the biochemical IC_50_ of compound 1f against M^pro^ lies in the high double-digit micromolar range (61.77 µM), which is modest compared to advanced covalent or optimized clinical candidates, it is important to explicitly classify 1f as an early-stage hit scaffold rather than a finalized lead. Hit compounds inherently possess higher micromolar potencies but offer structural tractability and novel scaffolding for future modification. Crucially, when evaluated in a live cellular virus assay, 1f demonstrated a significantly enhanced antiviral potency with an IC_50_ of 16.02 µM alongside a clean safety profile (CC_50_ >50 µM, selectivity index of 3.12). This roughly 4-fold increase in cellular potency compared to the biochemical assay is notable and highlights several advantageous properties: it points toward favorable intracellular accumulation, potential multi-targeting behavior, or synergistic modulation of host pathways involved in viral replication that are absent in isolated enzyme assays. Therefore, achieving low-micromolar antiviral efficacy paired with low cellular toxicity establishes compound 1f as a biologically validated, highly tractable starting point for future structure–activity relationship (SAR) driven hit-to-lead optimization. Taken together, the consistent antiviral activity of 1f across biochemical and cellular assays highlights its potential as a tractable hit scaffold targeting SARS-CoV-2 M^pro^.

### 2.2. *In silico* investigations

#### 2.2.1. Docking results

This section aims to explore the potential binding mode of compound 1f within the active site of the SARS-CoV-2 M^pro^ enzyme. As revealed from the enzyme assay, compound 1f was found to act through a non-covalent mechanism. Accordingly, a non-covalent docking approach using Autodock Vina was applied to conduct the docking study. Interestingly, compound 1f showed a good docking score of −14.1 kcal mol^−1^, while the reference compound showed a docking score of −17.6 kcal mol^−1^.

As illustrated in Fig. 5, compound 1f engaged in multiple interactions with critical residues of the SARS-CoV-2 M^pro^ enzyme. Specifically, it formed seven hydrogen bonds with essential residues, including Leu141, Asn142, Cys145, and His164. Additionally, compound 1f established hydrophobic interactions with His41, Met49, Glu166, and Asp187. These results highlight the compound's potential as a SARS-CoV-2 M^pro^ inhibitor and prompt us to conduct further *in silico* studies utilizing molecular dynamics.

**Fig. 5:**
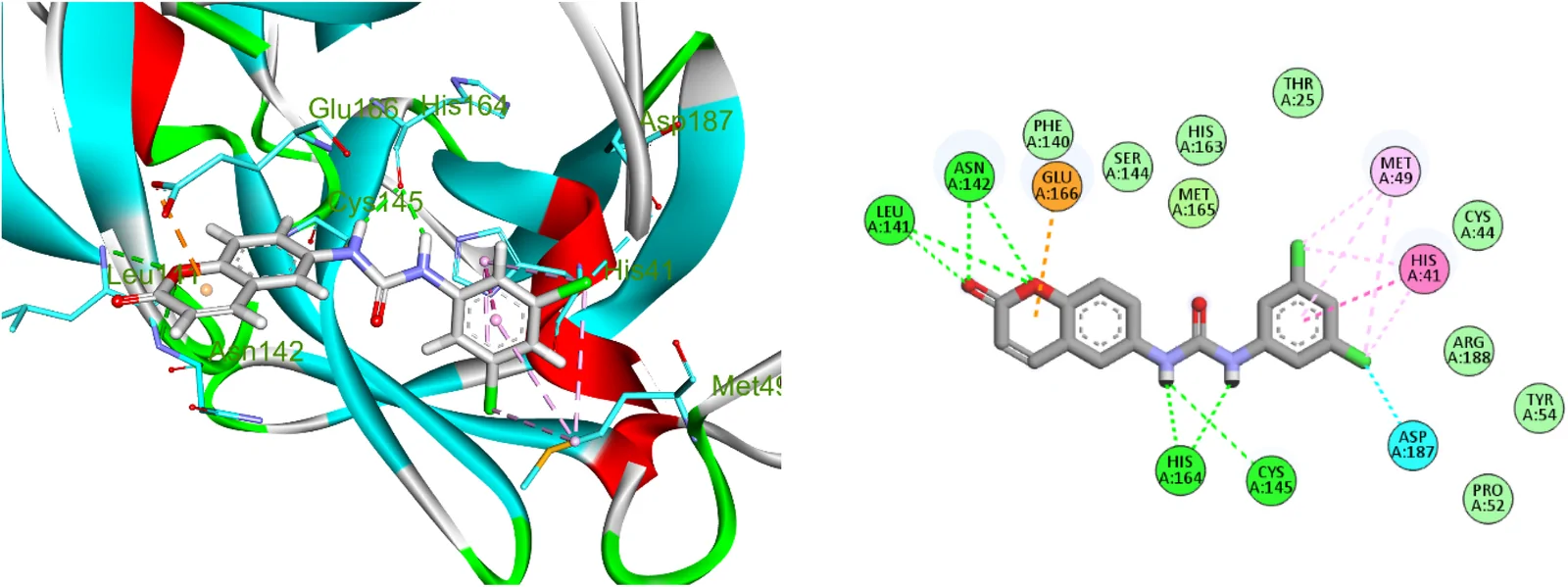
3D and 2D interaction diagram of SARS-CoV-2 M^pro^ bound to 1f (PDB ID: 7L0D).

#### 2.2.2. Molecular dynamics

In the current study, molecular dynamics (MD) simulations were employed to investigate ligand–target interactions *in silico*, offering valuable insights into the dynamic behavior of biological systems and enabling precise quantification of binding affinity in ligand–protein complexes. Two systems were subjected to MD simulations: the docked complex of 1f with SARS-CoV-2 M^pro^ (generated *via* molecular docking) and the crystallographic complex of SARS-CoV-2 M^pro^ with the non-covalent inhibitor ML188. To establish a baseline for comparison, simulations were performed on the apo form (unbound state) of SARS-CoV-2 M^pro^, to allow for a systematic evaluation of each ligand's impact on the structural stability and dynamic properties of the viral protease.

As demonstrated by Fig. 6, the two inhibitors, 1f and ML188, were able to stabilize the SARS-CoV-2 M^pro^ enzyme, as indicated by their lower RMSD values compared to the RMSD value of Apo SARS-CoV-2 M^pro^. The SARS-CoV-2 M^pro^-1f complex had an average RMSD value of 1.97 Å, which was in the middle between the RMSD values of SARS-CoV-2 M^pro^-ML188 (1.41 Å), and the RMSD of the apo SARS-CoV-2 M^pro^, which reached 3.37 Å.

**Fig. 6:**
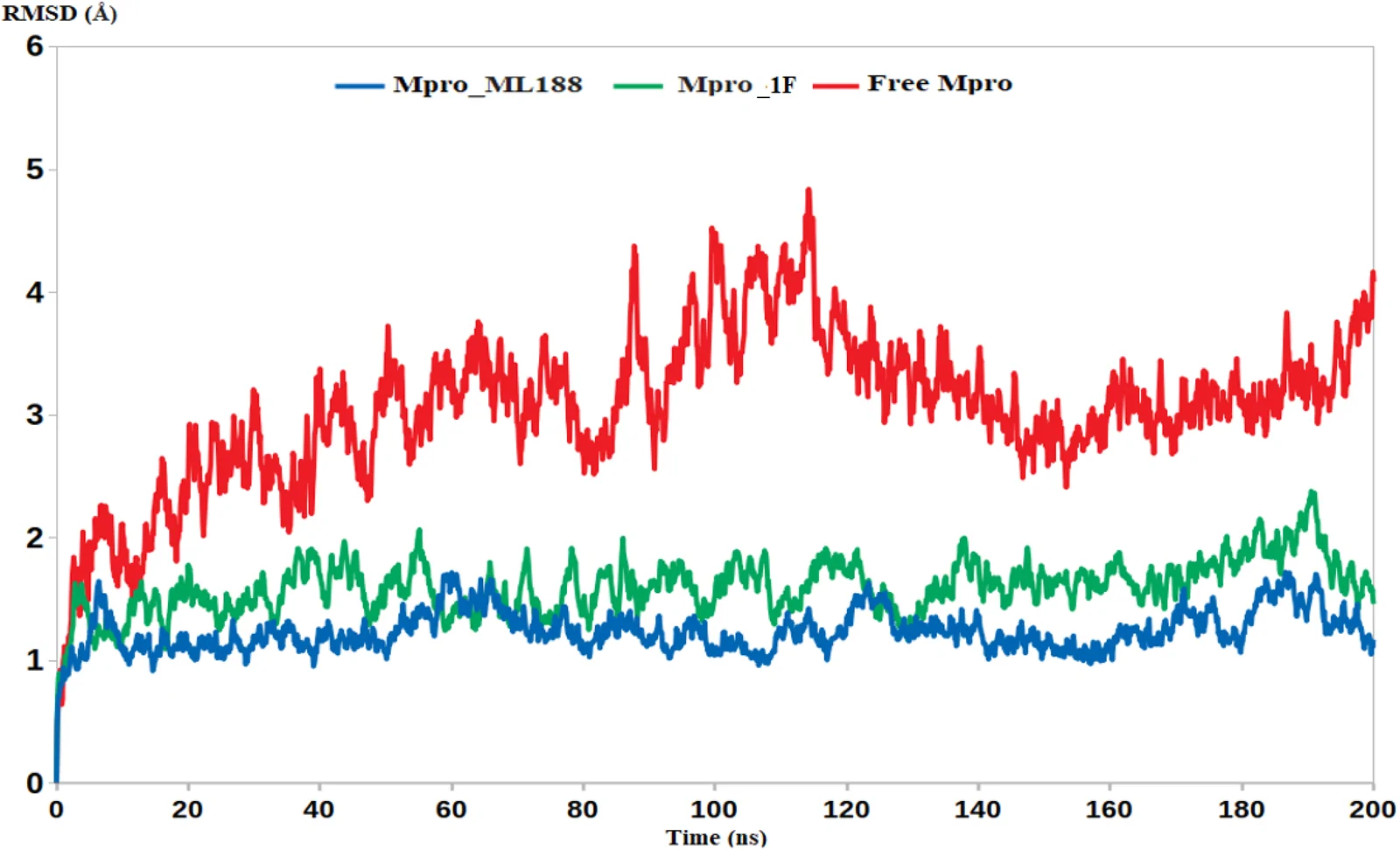
The RMSD of the Apo SARS-CoV-2 (red), SARS-CoV-2-1f (green), and SARS-CoV-2-ML188 (blue).

Similar results were obtained from the analysis of the RMSF values, in which the average root mean square fluctuation of C complex residues was about 1.98 Å. On the other hand, the residues of both SARS-CoV-2 M^pro^-ML188 and the apo SARS-CoV-2 M^pro^ showed average RMSF values of 1.37 and 3.91 Å, respectively. The findings support that 1f has a potential inhibitory activity against the SARS-CoV-2 virus through inhibition of the M^pro^ enzyme, Fig. 7.

**Fig. 7:**
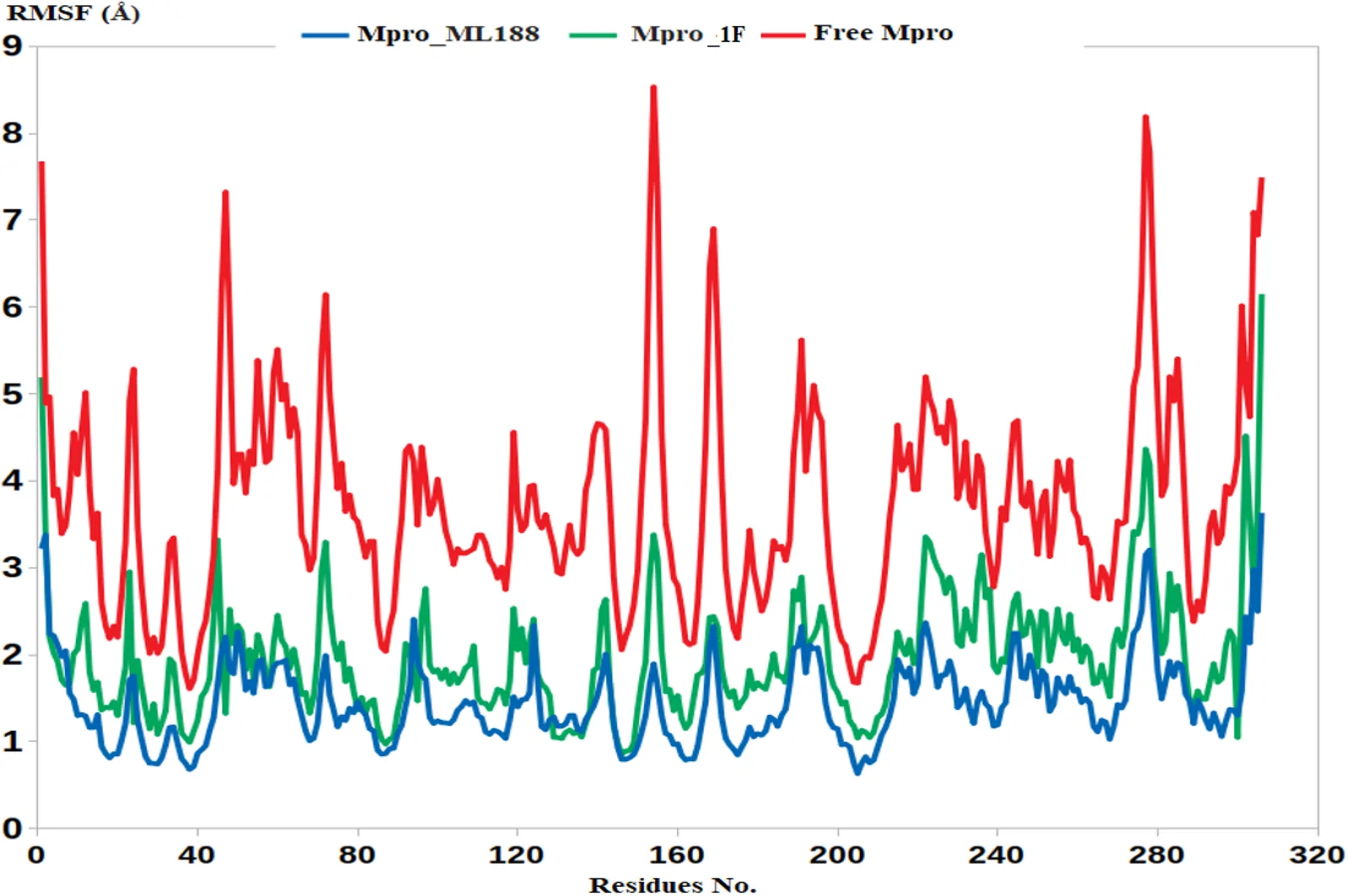
The RMSF of the Apo SARS-CoV-2 M^pro^ (red), SARS-CoV-2 M^pro^—1f (green), and SARS-CoV-2-ML188 (blue).

To further support our molecular dynamics results, the gmx_mmpbsa package was used to calculate the binding free energies resulting from the binding of 1f and ML188 to the SARS-CoV-2 M^pro^ enzyme. The generated trajectories from the production stage were used to calculate all forms of binding free energy. These energy types include electrostatic energy, van der Waals energy, polar solvation energy, and SASA energy. All the previous types of energy were calculated for the two complexes containing 1f and ML188 bound to SARS-CoV-2 M^pro^, Table 3. Notably, the calculated binding free energy for SARS-CoV-2 M^pro^—1f was favorable but higher than that for SARS-CoV-2 M^pro^-ML188. 1f achieved a binding free energy of −208.1 ± 2.1 (kJ mol^−1^), whereas ML188 achieved −306.8 ± 3.4 (kJ mol^−1^) upon binding to SARS-CoV-2 M^pro^. These binding free energy calculations corroborate the molecular docking results and substantiate the overall *in silico* findings.

**Table 3:** Summary of the interaction energies and the binding free energy for both complexes

Complex	Δ*E*_Binding (kJ mol_^−1^_)_	Δ*E*_Electrostatic (kJ mol_^−1^_)_	Δ*E*_van der Waals (kJ mol_^−1^_)_	Δ*E*_Polar solvation (kJ mol_^−1^_)_	SASA _(kJ mol_^−1^_)_
SARS CoV-2 M^pro^—1f	−208.1 ± 2.1	−131.6 ± 1.9	−109.8 ± 2.5	65.4 ± 1.5	−32.1 ± 0.9
SARS CoV-2 M^pro^—ML188	−306.8 ± 3.4	−201.5 ± 2.8	−183.3 ± 3.7	117.9 ± 2.7	−39.9 ± 1.0

## 3. Conclusions

This study focused on the biological evaluation of 19 coumarin derivatives as potential inhibitors of SARS-CoV-2 M^pro^. All molecules were screened for their inhibitory activity against M^pro^. In particular, coumarin 1f (AKE-9306) emerged as the most effective candidate, with an IC_50_ value of 61.77 µM. Enzyme kinetics analysis through Michaelis–Menten and Lineweaver–Burk plots revealed that compound 1f acts through a mixed-type inhibition mechanism with a preference for the free enzyme. 1f exhibited significant antiviral effects against SARS-CoV-2, achieving a 68.5% reduction in viral replication at 25 µM while preserving cell viability. A dose–response study determined an IC_50_ value of 16.02 µM and a selectivity index of 3.12. Immunofluorescence analysis validated the concentration-dependent suppression of nucleocapsid (N) protein expression. The superior efficacy observed in cellular assays suggests enhanced intracellular accumulation and potential modulation of host pathways, reinforcing 1f's promise as a non-covalent hit targeting SARS-CoV-2 M^pro^. Molecular docking and dynamics simulations provided significant insights into the binding mode, revealing multiple hydrogen bonds with critical residues, including Leu141, Asn142, Cys145, and His164, as well as hydrophobic interactions with His41, Met49, Glu166, and Asp187. The RMSD and RMSF values from molecular dynamics simulations confirmed the stabilizing effect of compound 1f on SARS-CoV-2 M^pro^. Taken together, these findings establish the arylurea–coumarin chemotype as a structurally novel, non-covalent hit scaffold with promising cellular activity, providing a tractable starting point for future hit-to-lead optimization targeting SARS-CoV-2 M^pro^.

## 4. Experimental

### 4.1. Chemicals and reagents

The coumarin derivatives 1a–p and 2a–c discussed in this study were synthesized as previously reported.^39^ All other reagents are from Sigma-Aldrich (MA, USA), otherwise stated.

### 4.2. Biological evaluations

#### 4.2.1. SARS-CoV-2 M^pro^ assay

##### 4.2.1.1. Cloning, protein expression, and purification

The gene encoding the SARS-CoV-2 M^pro^ from the Wuhan-Hu-1 isolate was ordered from Integrated DNA Technologies (IDT, USA) and cloned into the pET21a(+) vector. This construct was transformed into *Escherichia coli* BL21(DE3) chemically competent cells (Cat# CP111, Enzynomics, Korea). A single colony was inoculated into 4 mL of Luria Broth (LB) medium (Millipore, USA) supplemented with ampicillin (0.1 mg mL^−1^; Cat# 14 417, Cayman Chemical, USA) and cultured overnight at 37 °C with shaking at 180 rpm. The pre-culture was subsequently diluted 1 : 100 into 400 mL of fresh LB medium containing the same antibiotic concentration and incubated at 37 °C until the optical density at 600 nm (OD_600_) reached 0.4–0.5. Protein expression was induced with 0.5 mM isopropyl β-d-1-thiogalactopyranoside (IPTG; Cat# Sc-202185B, Santa Cruz Biotechnology, Inc., USA), followed by an additional 3-hours incubation at 37 °C, 180 rpm. Cells were harvested by centrifugation at 4000 rpm for 30 minutes at 4 °C and resuspended in BugBuster Master Mix (Cat# 71 456-4, Merck, USA), following the manufacturer's protocol. After lysis, the mixture was centrifuged at 14 000 rpm for 10 minutes at 4 °C to remove cell debris. The resulting supernatant was incubated for 1 hour at 4 °C with Ni-NTA Agarose beads (Lot# 175 039 928, Qiagen, Germany) on a rotator. The bead-bound protein was subsequently loaded onto an Econo-Pac^®^ chromatography column (Cat# 7 321 010, BIO-RAD, USA) for immobilized metal affinity chromatography (IMAC) purification. The column containing the beads was washed with Buffer A, composed of 50 mM Tris–HCl (pH 8.0; Cat# TR2016-050-74, Biosesang, Korea), 300 mM sodium chloride (Cat# SR2007-050-00, Biosesang, Korea; Lot# S0723Z13PU), 1 mM dithiothreitol (DTT; Cat# D2019, Biosesang, Korea), and 20 mM imidazole (Cat# IR2003-050-80, Biosesang, Korea). Elution was performed using buffer B, which contained the same components as buffer A, with 300 mM imidazole and 10% glycerol (Cat# GR1018-100-00, Biosesang, Korea). The eluted fractions were concentrated using Vivaspin^®^ centrifugal filter units (MWCO 10,000). Finally, Fast Protein Liquid Chromatography (FPLC) using a HiLoad™ 16/60 Superdex™ 200 pg column (Cat# 17-1069-01, GE Healthcare, Sweden) was employed for desalting to ensure protein purity. Protein purity was confirmed by SDS-PAGE using 4–15% Mini-PROTEAN^®^ TGX™ precast protein gels (Cat# 4 561 086, BIO-RAD, USA). Protein concentration was determined using both the Pierce™ BCA Protein Assay Kit (Cat# 23 225, Thermo Scientific, USA) and the NanoDrop ND-1000 UV/VIS spectrophotometer (Version 3.8.1, Thermo Scientific, USA), based on absorbance at 280 nm.

##### 4.2.1.2. Enzymatic assays

For the enzymatic inhibition study of SARS-CoV-2 M^pro^, a fluorogenic Förster Resonance Energy Transfer (FRET) substrate (DABCYL-KTSAVLQ ↓ SGFRKME-EDANS, Cat# 79 952, BPS Bioscience, USA) was employed. The cleavage of this substrate by the protease liberates the EDANS fluorophore, leading to fluorescence emission, thereby facilitating kinetic measurement of proteolytic activity. EDANS was purchased from Selleckchem (Houston, TX, USA) and used as a fluorescence calibration standard to convert relative fluorescence units (RFU) into product concentrations for the determination of kinetic parameters, including *K*_m_ and *K*_cat_.

The reaction system (total volume: 100 µL) was assembled in a sequential order, beginning with the buffer, followed by the addition of the enzyme and inhibitors, and finally the substrate, as previously described.^44,45^ All inhibitors were dissolved in dimethyl sulfoxide (DMSO; Cat# D8418, Sigma-Aldrich, USA) to prepare 10 mM stock solutions. The enzymatic reaction was carried out using 370 nM SARS-CoV-2 M^pro^ and 20 µM FRET substrate in 20 mM Tris–HCl buffer (pH 7.4) (Cat# TR2016-050-74, Biosesang, Korea). Inhibitors were tested at a final concentration of 100 µM. The reaction mixtures were incubated at 25 °C for 30 minutes, and fluorescence signals were recorded every 60 seconds using a Multi-Detection Microplate Reader (Synergy HT, BioTek Instruments, USA) with excitation and emission wavelengths set at 360/40 nm and 460/40 nm, respectively. The experimental design included appropriate negative controls (no inhibitor) and positive controls (using known M^pro^ inhibitors) to validate assay performance. To determine the half-maximal inhibitory concentration (IC_50_), inhibitor concentrations were tested by two-fold serial dilution from 200 µM to 6.25 µM for 1f, and by three-fold serial dilution from 100 µM to 0.4 µM for the positive control Baicalein. The initial reaction velocities were derived from the linear range of fluorescence increase and plotted against inhibitor concentrations. IC_50_ values were calculated by nonlinear regression fitting using GraphPad Prism software version 10.1.1 (GraphPad Software, USA).

#### 4.2.2. Michaelis–Menten kinetics assay

Enzyme kinetic experiments were performed at a fixed SARS-CoV-2 M^pro^ concentration of 370 nM using substrate concentrations of 5, 10, 20, 30, and 40 µM, across inhibitor concentrations of 1f at 0, 35, 70, 140, and 280 µM. Initial velocities were calculated from the linear phase of substrate hydrolysis. The untransformed initial-velocity data were globally fitted in GraphPad Prism 10.3.1 to competitive, noncompetitive, uncompetitive, and mixed-inhibition models. Among the models tested, the mixed-inhibition model best described the data and was selected to characterize the inhibition mechanism. The fitted inhibition constant (*K*_i_) and *α* value from this model were used to characterize the inhibition mechanism.

#### 4.2.3. Cytotoxicity assay

The cytotoxicity of AKE-9306 (1f) was evaluated in IMR-90 human lung fibroblast cells. IMR-90 cells were obtained from the American Type Culture Collection (ATCC, Manassas, VA, USA) and cultured in DMEM + GlutaMAX™ (Gibco, Grand Island, NY, USA) medium supplemented with 10% fetal bovine serum (Gibco) and 100 U mL^−1^ penicillin–streptomycin (Gibco). Cells were maintained at 37 °C in a humidified incubator containing 5% CO_2_. For the viability assay, IMR-90 cells were seeded (5 × 10^4^ cells per well) in 96-well plates and incubated for 24 h to allow cell attachment. The cells were then treated with 1f at concentrations ranging from 3.12 to 100 µM for 24 h, while control wells received an equivalent volume of DMSO. Cell viability was determined using the EZ-Cytox assay kit (DoGenBio, Seoul, South Korea) according to the manufacturer's instructions. Briefly, EZ-Cytox reagent was added to each well and incubated for 1.5 h at 37 °C. Absorbance was then measured at 450 nm using a Synergy HT multi-detection microplate reader (BioTek Instruments, Inc., Winooski, VT, USA). Cell viability was calculated relative to the DMSO-treated control group and expressed as a percentage.

#### 4.2.4. Live virus assay

##### 4.2.4.1. Virus and cells

Vero and Vero E6 cells, obtained from American Type Culture Collection (ATCC CCL-81, CRL-1586), were maintained at 37 °C in a 5% CO_2_ atmosphere using Dulbecco's Modified Eagle Medium (DMEM; #LM001-01, Welgene), supplemented with 10% heat-inactivated fetal bovine serum (FBS, Gibco, #A5670801) and 1× antibiotic–antimycotic (#15240062, Gibco). The ancestral SARS-CoV-2 (βCoV/KOR/KCDC03/2020) was provided by National Culture Collection for Pathogens (NCCP, #43326), and propagated in Vero E6 cells. Viral titers were determined by plaque assay. All experiments with SARS-CoV-2 were conducted at Institut Pasteur Korea in compliance with the guidelines of Korea National Institute of Health (kNIH), using enhanced Biosafety Level 3 (BSL-3) containment procedures in laboratories approved for use by Korea Disease Control and Prevention Agency (kDCA).

##### 4.2.4.2. Immunofluorescence assay of SARS-CoV-2 infection

Vero cells were seeded at a density of 1.2 × 10^4^ cells per well in 384-well black µClear plates (#781906, Greiner Bio-One) containing DMEM supplemented with 2% fetal bovine serum (FBS, #A5670801, Gibco) and 1× antibiotic–antimycotic solution (#15240062, Gibco). After 24 hours of incubation, cells were treated with test compounds at final concentrations ranging from 0.1 to 50 µM to generate ten-point dose–response curves. The plates were then transferred into a biosafety level 3 (BSL-3) facility, where infection with the ancestral strain of SARS-CoV-2 was carried out at a multiplicity of infection (MOI) of 0.1. Following 24 hours post-infection, cells were fixed with 4% paraformaldehyde and permeabilized using 0.25% Triton X-100. Antiviral efficacy was assessed by immunofluorescent detection of the viral nucleocapsid (N) protein. Fluorescent imaging was performed using the Operetta CLS High-Content Imaging System (PerkinElmer, MA, USA), and images were analyzed with Columbus software (PerkinElmer, Inc.) to determine total cell counts and infection rates. In each assay plate, infection levels were normalized against the mock-infected control (positive control) and 0.5% DMSO-treated wells (negative control). Dose–response curves (DRCs) were generated using a four-parameter sigmoidal model in GraphPad Prism Software (GraphPad Software, San Diego, CA), from which IC_50_ and CC_50_ values were calculated. All measurements were performed in duplicate, and assay quality was monitored using the *Z*′-factor and percent coefficient of variation (%CV).

##### 4.2.4.3. Statistical analysis

Statistical analyses were performed using GraphPad Prism (Version 10.2.1; GraphPad Software, Inc., La Jolla, CA). Values are presented in the bar graphs as the mean ± SD from two independent experiments.

### 4.3. Molecular modeling

#### 4.3.1. Molecular docking

Autodock Vina and M. G. L. tools were utilized in the docking study, whereas Discovery Studio was employed for results visualization. AutoDock Vina utilizes an Amber force field and a united-atom scoring function. Additionally, Vina utilizes a global optimization algorithm, specifically a gradient-based local search genetic algorithm, to predict how small molecules bind to their targets.^46^ Using MGL tools, ligands 1f and ML188) and SARS-CoV-2 M^pro^ (PDB ID: 7L0D) were prepared and converted to pdbqt format. The active site of SARS-CoV-2 M^pro^ was defined using the co-crystallized ligand's binding coordinates, with a grid box of 22 × 22 × 22 Å in the *x*, *y*, and *z* dimensions. For validation, the co-crystallized reference ligand (ML188) was redocked into its active site, yielding an RMSD of 0.71 Å between the docked pose and the original crystal structure. Finally, the results were analyzed using docking scores and binding interactions retrieved from the 2D interaction diagrams.

#### 4.3.2. Molecular dynamics

Three 200-ns molecular dynamics (MD) simulations were performed using GROMACS 2023.2.^47^ The retrieved docking coordinates of the SARS-CoV-2 M^pro^ enzyme in complex with the target compound 1f and compound ML188, in addition to apo M^pro^, were used as input structures for the MDS. Receptor topologies were generated with GROMACS's PDB2gmx tool, while ligand topologies were prepared using the Acpype server, both under the AMBER force field.^48,49^ Simulation protocols were performed as previously described.^11^

## Author contributions

Ashraf K. El-Damasy: conceptualization, data curation, funding acquisition, investigation, methodology, project administration, resources, visualization, writing – original draft, writing – review and editing; Md Sofequl Islam Mukim: data curation, formal analysis, funding acquisition, investigation, methodology, resources, writing – original draft; Sangeun Jeon: data curation, methodology, resources, visualization; Seungtaek Kim: data curation, methodology, resources, validation; Mahmoud A. El Hassab: data curation, software, writing – original draft, Suliman A Almahmoud: formal analysis, investigation, methodology; Eun-Kyoung Bang: formal analysis, investigation, validation; Wagdy M. Eldehna: formal analysis, investigation, writing – original draft, writing – review and editing; Dae-Geun Song: funding acquisition, investigation, project administration, resources, supervision, writing – review and editing; Gyochang Keum: funding acquisition, investigation, project administration, supervision, writing – review and editing.

## Conflicts of interest

The authors declare no conflicts of interest.

## Supplementary Material

RA-OLF-D6RA07298D-s001

## Data Availability

All relevant data are within the manuscript and its supplementary information (SI). Raw datasets, including IC_50_ curves, enzyme kinetics, docking poses, and molecular dynamics trajectories, will be deposited upon acceptance. Supplementary information is available. See DOI: https://doi.org/10.1039/d6ra07298d.
