# Supplementary material for: Evaluation of 6-substituted coumarins as SARS-CoV-2 main protease inhibitors: enzymatic, cellular, and computational insights
Source: RSC Adv. 2026 Oct 5;16(46):60280–91. doi: 10.1039/d6ra07298d (PMC13637745; doi:10.1039/d6ra07298d)
Supplement: Supplementary file 1 [file RA-016-D6RA07298D-s001.pdf]

Supplementary Information (SI) for RSC Advances.
This journal is © The Royal Society of Chemistry

Supplementary Data

Evaluation of 6-Substituted Coumarins as SARS-CoV-2 Main Protease Inhibitors: Enzymatic, Cellular, and Computational Insights

Ashraf K. El-Damasy[a,b,*,†], Md Sofequl Islam Mukim[c,d,†], Sangeun Jeon[e], Mahmoud A. El Hassab[f], Suliman A Almahmoud[g], Eun-Kyoung Bang[a], Wagdy M. Eldehna[h,*], Dae-Geun Song [c,d,*], Seungtaek Kim[e*], Gyochang Keum [a,i*]

[a] *Biomedical Research Division, Korea Institute of Science and Technology (KIST), Seoul 02792, Republic of Korea*
[b] *Department of Medicinal Chemistry, Faculty of Pharmacy, Mansoura University, Mansoura 35516, Egypt*
[c] *Center for Natural Product Systems Biology, KIST Gangneung Institute of Natural Products, Gangneung, 25451, Republic of Korea*
[d] *Natural Product Applied Science, KIST School, University of Science and Technology, Gangneung 25451, Republic of Korea*
[e] *Zoonotic Virus Laboratory, Institut Pasteur Korea, Seongnam, Republic of Korea*
[f] *Department of Medicinal Chemistry, Faculty of Pharmacy, King Salman International University (KSIU), South Sinai 46612, Egypt*
[g] *Department of Pharmaceutical Chemistry and Pharmacognosy, College of Pharmacy, Qassim University, Buraydah51452, Saudi Arabia*
[h] *Department of Pharmaceutical Chemistry, Faculty of Pharmacy, Kafrelsheikh University, Kafrelsheikh, P.O. Box 33516, Egypt*
[i] *Division of Bio-Medical Science and Technology, KIST School, University of Science and Technology, Seoul 02792, Republic of Korea*

---

* Corresponding authors' E-mail addresses: ph_karem2000@mans.edu.eg (A. K. El-Damasy), seungtaek.kim@ip-korea.org (Seungtaek Kim), dsong82@kist.re.kr (Dae-Geun Song), gkeum@kist.re.kr (G. Keum), wagdy2000@gmail.com (W. M. Eldehna).

† Co-first authors.

Supplementary Table 1: Primary Screening of **AKE-9306 (1f)** in Vero cells.

**Primary Screening**				
**Sl No**	**Name**	**Conc.**	**Inhibition%**	**Viability %**
1	AKE-9306/1f	25.0	68.5	84.0
		5.0	-3.4	91.6
2	Remdesivir	25.0	95.4	92.6
		3.1	7.1	84.9

Vero E6 cells were infected with ancestral SARS-CoV-2 at a multiplicity of infection (MOI) of 0.1. Cell viability was assessed 72 hours post-infection.

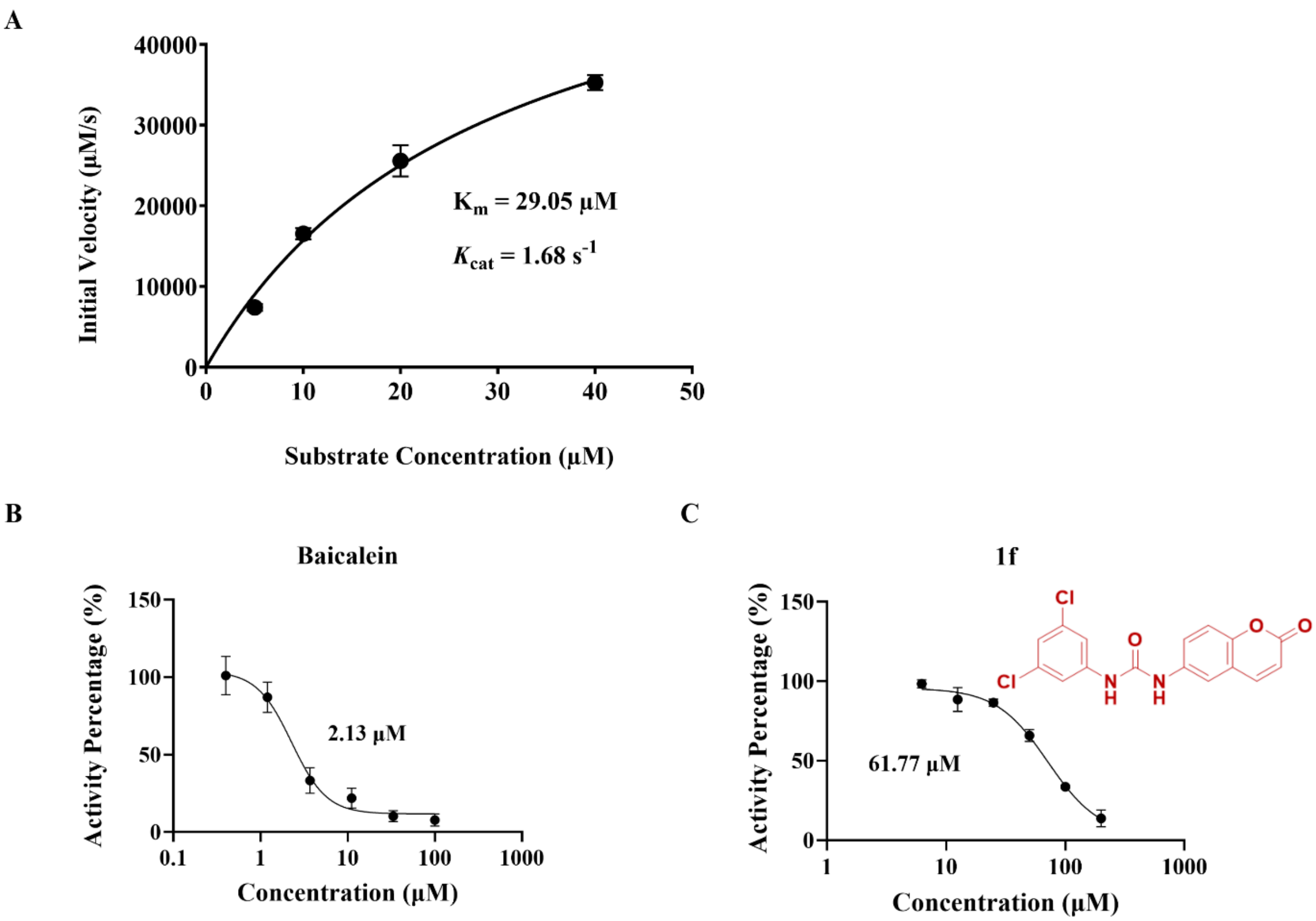

Supplementary Figure S1. Functional validation of M^{pro} and IC_{50} determination. (A) Michaelis–Menten kinetic analysis of purified SARS-CoV-2 M^{pro} using a FRET substrate, showing an apparent K_m of 29.05 μM and k_{cat} of 1.68 s^{-1}. (B) Dose-dependent inhibition of M^{pro} by Baicalein (IC_{50}= 2.13 μM), and (C) compound **1f** (AKE-9306) (IC_{50}= 61.77 μM). Data are presented as the mean ± SD.
